# Functional connectivity of the medial prefrontal cortex related to mindreading abilities

**DOI:** 10.1093/texcom/tgac032

**Published:** 2022-07-31

**Authors:** Marine Le Petit, Francis Eustache, Joy Perrier, Vincent de La Sayette, Béatrice Desgranges, Mickaël Laisney

**Affiliations:** Normandie Univ, UNICAEN, PSL Université Paris, EPHE, INSERM, U1077, CHU de Caen, Centre Cyceron, Neuropsychologie et Imagerie de la Mémoire Humaine, 14000 Caen, France; Normandie Univ, UNICAEN, PSL Université Paris, EPHE, INSERM, U1077, CHU de Caen, Centre Cyceron, Neuropsychologie et Imagerie de la Mémoire Humaine, 14000 Caen, France; Normandie Univ, UNICAEN, PSL Université Paris, EPHE, INSERM, U1077, CHU de Caen, Centre Cyceron, Neuropsychologie et Imagerie de la Mémoire Humaine, 14000 Caen, France; Normandie Univ, UNICAEN, PSL Université Paris, EPHE, INSERM, U1077, CHU de Caen, Centre Cyceron, Neuropsychologie et Imagerie de la Mémoire Humaine, 14000 Caen, France; Normandie Univ, UNICAEN, PSL Université Paris, EPHE, INSERM, U1077, CHU de Caen, Centre Cyceron, Neuropsychologie et Imagerie de la Mémoire Humaine, 14000 Caen, France; Normandie Univ, UNICAEN, PSL Université Paris, EPHE, INSERM, U1077, CHU de Caen, Centre Cyceron, Neuropsychologie et Imagerie de la Mémoire Humaine, 14000 Caen, France

**Keywords:** functional magnetic resonance imaging, mirror neurons, prefrontal cortex, social cognition, theory of mind

## Abstract

The medial prefrontal cortex is a key region of mindreading belonging to the mentalizing system, a set of brain areas underlying mental state inference based on reasoning on social concepts. The aim of this study was to characterize the functional connectivity between regions involved in mindreading and to highlight the processes it underpins, focusing on the dorsal and ventral parts of the medial prefrontal cortex. We analyzed resting-state functional magnetic resonance imaging of 56 healthy volunteers, to study the relationship between mindreading abilities and functional connectivity of the medial prefrontal cortex. Cognitive mindreading performances were correlated with connectivity between the medial prefrontal cortex and frontal regions involved in the regulation of the salience of one’s own mental contents, with a distinction between the dorsal part connected to regions subtending inhibition processes and the ventral part to emotional regions. Affective mindreading performances were negatively correlated with negative connectivity of the ventro- and dorsomedial prefrontal cortex with sensorimotor regions belonging to the mirror neuron system subtending the simulation of mental states. These findings suggested a role of the medial prefrontal cortex to decrease the salience of one’s own mental content and in the antisynchronous interaction between the mentalizing and mirror neurons systems.

## Introduction

The ability to attribute mental states to oneself or others is a key aspect of social cognition. This ability is referred to as Theory of mind (Premack and Woodruff 1978) or *mindreading* (Apperly 2010). Mindreading comprises cognitive and affective components that are partly dissociable at the neural level (Shamay-Tsoory and Aharon-Peretz 2007). *Cognitive mindreading* refers to the ability to understand, infer or reason about thoughts, beliefs, knowledge, or intentions without taking emotional aspects into account. *Affective mindreading* is defined as the processing of affective mental states such as emotions and feelings (Shamay-Tsoory and Aharon-Peretz 2007; Abu-Akel and Shamay-Tsoory 2011). As emphasized by Happé et al. (2017), the ability to adopt another person’s perspective and to distinguish between one’s own and others’ perspectives is an important requirement for correctly inferring mental states. Furthermore, it is essential to understand that one’s own perspective (e.g. visual perspective) is not the same as others’. The ability to inhibit one’s own perspective, in favor of the perspective of others, is therefore an important prerequisite for mindreading (Samson et al. 2007; Samson 2009).

The brain areas associated with mindreading processes have been clearly identified. They form several networks, notably the so-called mentalizing system (MZS), which is assumed to support reasoning on mental states. The MZS encompasses the medial prefrontal cortex (mPFC), temporoparietal junction (TPJ), precuneus, posterior cingulate cortex, and temporal poles, which all play an important role (Schurz et al. 2014).

The mPFC is frequently identified as a core region in mindreading studies (Frith and Frith 2001), but the processes it subtends and its specific involvement in mindreading (Otti et al. 2015; Boccadoro et al. 2019) are still subject to debate, owing to the large portion of the brain it represents and the number of cognitive processes in which it is involved. Because the dorsal and ventral parts of the mPFC have been consistently distinguished on the basis of their involvement in mindreading processes, some authors assumed a dorso-ventral gradient in the mPFC (Amodio and Frith 2006). The more dorsal part of the mPFC (dmPFC) was associated with the executive processes that underpin mindreading, such as inhibition and decision-making (Isoda and Noritake 2013). In contrast, the more ventral part of the mPFC (vmPFC) was assumed to be linked to the monitoring of others’ emotional mental states (Amodio and Frith 2006). Besides, cognitive mindreading has been associated with the dmPFC and affective mindreading with the vmPFC (Abu-Akel and Shamay-Tsoory 2011). The mPFC has also been assumed to be involved in the distinction between self and others’ mental states. Mitchell et al. (2005) observed differential responses by the two subparts of the mPFC: the vmPFC appeared to be more specifically involved in the attribution of mental states to oneself or to others regarded as similar to the self, whereas the dmPFC was more elicited by the attribution of mental states to others regarded as nonsimilar to the self. Further evidence of the vmPFC’s involvement in self-referential processing comes from studies by Northoff et al. (2006) and D’Argembeau et al. (2007). In parallel, the dmPFC is assumed to play a role in the decoupling of different perspectives, enabling individuals to distinguish their own perspective from those of others (D’Argembeau et al. 2007; Isoda and Noritake 2013). In line with this, Gallagher and Frith (2003) suggested that the mPFC is involved in the attribution of abstract mental states when they are decoupled from reality, such as in false belief tasks. This suggestion has been strengthened by evidence of greater mPFC activity for false beliefs than for true ones (Döhnel et al. 2012).

While regions associated with mindreading have been extensively studied, less is known about their functioning as networks, including the connectivity of the mPFC. Task-based functional Magnetic Resonance Imaging (fMRI) studies have yielded some information, notably by the means of dynamic causal modeling analyses, highlighting the central role of the mPFC in the mindreading network. These studies suggested that subparts of the mPFC may differently contribute to its key role in mindreading (Li et al. 2014; Hill et al. 2017), by the means of positive and negative coupling between the mPFC and regions associated with mindreading (Schurz et al. 2020). Notably, a connectivity between the dmPFC and TPJ was highlighted (Li et al. 2014; Hill et al. 2017). In contrast, the vmPFC was connected to temporal regions and this connectivity was associated with emotional engagement during social interactions (Li et al. 2014). In regard with processes underlying mindreading, some authors proposed an integrative model in which the dmPFC and TPJ interact to integrate social information and transmit inputs to the vmPFC, allowing emotional values to be attributed to the different possible social choices (Hill et al. 2017).

The notion of resting-state cognition-oriented toward mindreading was suggested by responses to self-report questionnaires about brain activities (Diaz et al. 2013) and the substantial overlap of the default mode network—a well-known network evidenced during resting state in fMRI studies—with regions highlighted in mindreading tasks (Schilbach et al. 2008). Moreover, because tasks are not realized during the MRI scanning session, resting state fMRI allows for the use of long and complex tasks that better reflect everyday social situations to analyze the relationship between cognition and the brain’s functional architecture. It also allows for the observation of networks supporting different processes and the investigation of their interactions, in the course of the same analysis. However, to our knowledge, no study has yet investigated the resting-state functional connectivity of the dorsal and ventral mPFC in relation to mindreading. Additionally, whereas most mindreading studies have only investigated positive connectivity, some authors have stressed the need to take negative functional connectivity into account (Fox et al. 2005; Hampson et al. 2010; Liu et al. 2015). Studies investigating the relationships between the mPFC and attentional networks, which include regions associated with mindreading (Vossel et al. 2014), have frequently highlighted anticorrelations (Fox et al. 2005; Chen et al. 2017). Negative connectivity should therefore be considered when seeking to account for the mPFC’s connections with other regions that subtend mindreading processes. Besides, within the mindreading framework, authors have recently shown a keen interest in social interactions in context (Ibanez and Manes 2012). In the past, mindreading studies have often used tasks devoid of dynamic stimuli and social context. The use of a complex video task featuring everyday life situations where the protagonists’ interactions are placed in a dynamic social context would better reflect the processes that take place in real life.

This work aimed at clarifying the functional connectivity of both the dorsal and ventral mPFC associated with mindreading abilities measured out of the scanner, in order to (i) investigate synchronization mechanisms of brain regions with the mPFC associated with mindreading and (ii) question the theoretical proposition of a ventro-dorsal functional distinction within the mPFC; by determining the differential connectivities of each subpart of the mPFC related to mindreading. We therefore studied the positive and negative associations between the functional connectivity of both the dmPFC and vmPFC at rest and mental state attribution performances during the processing of social interactions in a social context among healthy volunteers. Based on previous research, we predicted that the connectivity of the mPFC with other regions of the MZS, notably the TPJ and temporal regions, as well as with the amygdala and IFG, would be associated with mindreading abilities. Given the different putative roles attributed to the ventral and dorsal parts of the mPFC, it can also be expected that this distinction would be reflected in the differential connectivities of the ventral and dorsal subparts.

## Materials and methods

### Participants

Participants were 60 healthy volunteers aged 20–75 years (31 women, mean age = 42.4 ± 17.0 years, mean years of education = 13.2 ± 2.4 years). Data from four participants were not included in analyses, as the functional images of two of them could not be preprocessed owing to a compatibility issue related to the preprocessing pipeline, while the other two had brain abnormalities revealed by the anatomical MRI. The inclusion of healthy volunteers representing a broad age range increased the variability of measures in order to enhance the power of the correlational analyses. As effects of age were not of interest, this variable was added as nuisance covariate. None of the participants, who were all French native speakers, had a history of alcoholism, psychiatric problems, or cognitive complaints, and none of the participants over 50 years of age had pathological global cognitive performances (Mattis Dementia Rating Scale mean score = 142.1 ± 1.8/144, minimum score = 138/144). Participants underwent anatomical and functional MRI (see Subsection 2.3) and a test assessing mindreading abilities (see Subsection 2.2). The experiment was approved by the regional ethics committee (CPP Nord-Ouest III), and all participants gave their written informed consent. The study was carried out in accordance with the Declaration of Helsinki.

### Assessment of mindreading abilities

Cognitive and affective mindreading abilities were assessed with the Movie for the Assessment of Social Cognition (MASC; Dziobek et al. 2006), translated into French at the Sainte-Justine University Hospital (Montreal). This computerized task is a 15-min movie featuring the social interactions of 4 protagonists at a dinner party. Understanding these social interactions requires the comprehension of mindreading components such as false belief, sarcasm, and white lies. The video is stopped 45 times, and participants are asked to answer 4-alternative forced-choice questions about the mental states of one of the protagonists. There are 18 questions about the protagonists’ intentions, 6 questions about their thoughts, 18 questions about the emotions of one of the protagonists, and 6 control questions assessing scene comprehension. A cognitive mindreading score was derived from the intentions items, and an affective mindreading score from the emotions items, but responses to the thoughts items were not analyzed because of their ambiguous nature. By getting closer to complex everyday social interactions, this test avoids ceiling effects in healthy participants and gives rise to a variability of performances within the group. The mean cognitive mindreading score was 10.7 ± 2.4 (range: 3–15), while the mean affective mindreading score was 11.6 ± 2.2 (range: 7–16). In addition, the mean control question score was 4.9 ± 0.9 (range: 3–6).

### fMRI data acquisition and preprocessing

#### fMRI data acquisition

Participants underwent anatomical and functional MRI. All images were acquired at the Cyceron centre (Caen, France) using a Philips (Eindhoven, The Netherlands) Achieva 3.0 T scanner.

First, a fast field echo sequence was used to acquire high-resolution *T*_1_-weighted (*T*_1_-w) anatomical images [3D-T1-FFE sagittal; SENSE factor = 2, time of repetition (TR) = 20 ms, time of echo (TE) = 4.6 ms, flip angle = 10°, 180 slices, with no gap between the slices, slice thickness = 1 mm, field of view (FOV) = 256 × 256 mm^2^, in-plane resolution = 1 × 1 mm^2^]. This was followed by a high-resolution *T*_2_-weighted spin echo anatomical scan (2D-T2-SE sagittal; SENSE factor = 2, TR = 5500 ms, TE = 80 ms, flip angle = 90°, 81 slices, with no gap between the slices, slice thickness = 2 mm, FOV = 256 × 256 mm^2^, in-plane resolution = 1 × 1 mm^2^). After the anatomical scanning, resting-state fMRI scans were acquired using an interleaved 2D T2^*^ EPI sequence (2D-T2^*^-FFE-EPI axial; SENSE factor = 2, TR = 2382 ms, TE = 30 ms, flip angle = 80°, 42 slices, with no gap between the slices, slice thickness = 2.8 mm, FOV = 224 × 224 mm^2^, in-plane resolution = 2.8 × 2.8 mm^2^, 280 volumes). The first 6 volumes were discarded, owing to saturation effects. Participants were equipped with earplugs and their head was stabilized with foam pads to minimize head motion. During the resting-state acquisition, they remained lying down with their eyes closed, but stayed awake.

The following 2 subsections are taken from the report of the fMRI data preprocessing performed with fMRIPrep and have been copied without being edited.

Results included in this manuscript come from preprocessing performed using fMRIPrep 1.5.4 (Esteban et al. 2019; https://fmriprep.org/ RRID:SCR_016216), which is based on Nipype 1.3.1 (Gorgolewski et al. 2011, 2017; https://nipype.readthedocs.io/, RRID:SCR_002502).

#### Anatomical data preprocessing

The *T*_1_-weighted (T1w) image was corrected for intensity nonuniformity with N4BiasFieldCorrection (Tustison et al. 2010), distributed with ANTs 2.2.0 (Avants et al. 2008, http://www.picsl.upenn.edu/ANTS/, RRID:SCR_004757), and used as T1w-reference throughout the workflow. The T1w-reference was then skull-stripped with a Nipype implementation of the antsBrainExtraction.sh workflow (from ANTs), using OASIS30ANTs as target template. Brain tissue segmentation of cerebrospinal fluid (CSF), white-matter (WM), and gray-matter (GM) was performed on the brain-extracted T1w using fast (FSL 5.0.9, http://fsl.fmrib.ox.ac.uk/fsl/fslwiki/, RRID:SCR_002823, Zhang et al. 2001). Volume-based spatial normalization to 2 standard spaces (MNI152NLin2009cAsym, MNI152NLin6Asym) was performed through nonlinear registration with antsRegistration (ANTs 2.2.0), using brain-extracted versions of both T1w reference and the T1w template. The following templates were selected for spatial normalization: ICBM 152 Nonlinear Asymmetrical template version 2009c [(Fonov et al. 2009), http://www.bic.mni.mcgill.ca/ServicesAtlases/ICBM152NLin2009, RRID:SCR_008796; TemplateFlow ID: MNI152NLin2009cAsym], FSL’s MNI ICBM 152 nonlinear 6th Generation Asymmetric Average Brain Stereotaxic Registration Model [(Evans et al. 2012), RRID:SCR_002823; TemplateFlow ID: MNI152NLin6Asym].

#### Functional data preprocessing

For each of the 1 blood-oxygen-level-dependent (BOLD) runs found per subject (across all tasks and sessions), the following preprocessing was performed. First, a reference volume and its skull-stripped version were generated using a custom methodology of fMRIPrep. Susceptibility distortion correction (SDC) was omitted. The BOLD reference was then co-registered to the T1w reference using flirt (FSL 5.0.9, Jenkinson and Smith 2001) with the boundary-based registration (Greve and Fischl 2009) cost-function. Co-registration was configured with 9 degrees of freedom to account for distortions remaining in the BOLD reference. Head-motion parameters with respect to the BOLD reference (transformation matrices, and 6 corresponding rotation and translation parameters) are estimated before any spatiotemporal filtering using mcflirt (FSL 5.0.9, Jenkinson et al. 2002). BOLD runs were slice-time corrected using 3dTshift from AFNI 20160207 (Cox and Hyde 1997), http://afni.nimh.nih.gov/afni/, RRID:SCR_005927). The BOLD time-series (including slice-timing correction when applied) were resampled onto their original, native space by applying the transforms to correct for head-motion. These resampled BOLD time-series will be referred to as preprocessed BOLD in original space or just preprocessed BOLD. The BOLD time-series were resampled into several standard spaces, correspondingly generating the following spatially normalized, preprocessed BOLD runs: MNI152NLin2009cAsym, MNI152NLin6Asym. First, a reference volume and its skull-stripped version were generated using a custom methodology of fMRIPrep. Automatic removal of motion artifacts using independent component analysis (ICA-AROMA, Pruim et al. 2015) was performed on the preprocessed BOLD on MNI space time-series after removal of non-steady state volumes and spatial smoothing with an isotropic, Gaussian kernel of 6 mm FWHM (full-width half-maximum). Corresponding “non-aggressively” denoized runs were produced after such smoothing. Additionally, the “aggressive” noise-regressors were collected and placed in the corresponding confounds file. Several confounding time-series were calculated based on the preprocessed BOLD: framewise displacement (FD), DVARS, and 3 region-wise global signals. FD and DVARS are calculated for each functional run, both using their implementations in Nipype (following the definitions by Power et al. 2014). The 3 global signals are extracted within the CSF, the WM, and the whole-brain masks. Additionally, a set of physiological regressors were extracted to allow for component-based noise correction (CompCor, Behzadi et al. 2007). Principal components are estimated after high-pass filtering the preprocessed BOLD time-series (using a discrete cosine filter with 128-s cut-off) for the 2 CompCor variants: temporal (tCompCor) and anatomical (aCompCor). tCompCor components are then calculated from the top 5% variable voxels within a mask covering the subcortical regions. This subcortical mask is obtained by heavily eroding the brain mask, which ensures that it does not include cortical GM regions. For aCompCor, components are calculated within the intersection of the aforementioned mask and the union of CSF and WM masks calculated in T1w space, after their projection to the native space of each functional run (using the inverse BOLD-to-T1w transformation). Components are also calculated separately within the WM and CSF masks. For each CompCor decomposition, the k components with the largest singular values are retained, such that the retained components’ time series are sufficient to explain 50% of variance across the nuisance mask (CSF, WM, combined, or temporal). The remaining components are dropped from consideration. The head-motion estimates calculated in the correction step were also placed within the corresponding confounds file. The confound time series derived from head motion estimates and global signals were expanded with the inclusion of temporal derivatives and quadratic terms for each (Satterthwaite et al. 2013). Frames that exceeded a threshold of 0.5-mm FD or 1.5 standardized DVARS were annotated as motion outliers. All resamplings can be performed with a single interpolation step by composing all the pertinent transformations (i.e. head-motion transform matrices, SDC when available, and co-registrations to anatomical and output spaces). Gridded (volumetric) resamplings were performed using antsApplyTransforms (ANTs), configured with Lanczos interpolation to minimize the smoothing effects of other kernels (Lanczos 1964). Non-gridded (surface) resamplings were performed using mri_vol2surf (FreeSurfer).

Before the start of each analysis, raw and preprocessed data were visually inspected for image artifacts and motion.

**Fig. 1:**
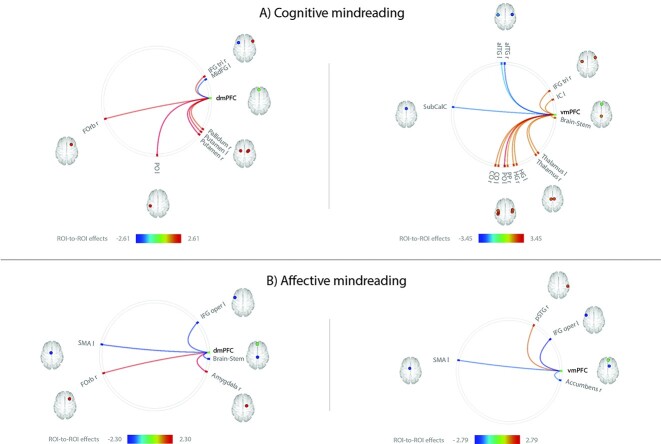
Significant correlations between the functional connectivity of the ventral and dorsal mPFC with brain regions of the Harvard-Oxford atlas and a) cognitive mindreading scores and b) affective mindreading scores. Statistical significance was thresholded at *P* < 0.05 uncorrected. Age was included as covariate of noninterest. Positive correlations are represented by hot colors ranging from yellow to red, and negative correlations by cold colors ranging from green to blue. aITG: Anterior inferior temporal gyrus; CO: Central opercular cortex; dmPFC: Dorsomedial prefrontal cortex; FOrb: Frontal orbital cortex; HG: Heschl’s gyrus; IC: Insular cortex; IFG oper: Inferior frontal gyrus pars opercularis; IFG tri: Inferior frontal gyrus pars triangularis; MidFG: Middle frontal gyrus; PO: Parietal operculum; PP: Planum polare; pSTG: Posterior superior temporal gyrus; SubCalC: Subcallosal cortex; vmPFC: Ventromedial prefrontal cortex; r: Right; l: Left.

### ROI-to-ROI analysis

Region of interest (ROI) analyses were performed using the MATLAB CONN Toolbox 15.g implemented in SPM12 (Whitfield-Gabrieli and Nieto-Castanon 2012). Images from the preprocessing step, such as structural, nonaggressively denoised AROMA functional images and GM, WM, and CSF images, were imported into the toolbox. A matrix composed of first-level confounds was created for each participant, containing the 6 movement parameters (trans and rot), the first 5 aCompCor ,and the first 5 tCompCor (Behzadi et al. 2007). The cognitive and affective mindreading scores were included as second-level covariates, as well as age, which was treated as a nuisance covariate. During the denoising step, signals from WM and CSF as well as the 16 first-level potential confounds were removed by linear regression. A bandpass filter was applied to each voxel (0.008–0.09 Hz), to reduce nonneurophysiological noise. Two 10-mm (radius) spherical ROIs centred on the coordinates extracted from a meta-analysis of brain imaging studies of mindreading (Schurz et al. 2014) were included as ROIs in the CONN toolbox. These ROIs were labeled dmPFC (peak coordinates in Montreal Neurological Institute space: x = −1, y = 54, z = 24) and vmPFC (peak coordinates in Montreal Neurological Institute space: x = 3, y = 51, z = −7). In addition, the 48 cortical and 21 subcortical ROIs from the FSL Harvard-Oxford maximum likelihood atlas except for the right and left Superior Frontal Gyrus and Medial Frontal Cortex that, respectively, overlapped with the dmPFC and vmPFC ROIs were included as ROIs in the first-level analysis. The functional connectivity between each pair of ROIs, defined as the Fisher-transformed correlation coefficient between BOLD time series of the two target ROIs, was computed, enabling the creation of ROI-to-ROI functional connectivity maps for each participant. Two second-level analyses were performed. For both analyses, the dmPFC and vmPFC spherical ROIs were separately used as seed regions, and the other ROIs as targets. The threshold for significance was set at *P* < 0.05 uncorrected. First, we calculated Pearson’s correlation coefficients between each mindreading score and the connectivity of the dmPFC and vmPFC with other regions of the atlas. Age was entered as a nuisance covariate. The interpretation of correlations between mindreading scores and the connectivity of both the dmPFC and vmPFC depended on the valence of their connectivity with the regions highlighted by the correlation analysis. Therefore, a complementary analysis consisted of one-sample *t*-tests testing for significant functional connectivity between the dmPFC or vmPFC, and the ROIs identified in the first analysis was conducted. Briefly, it was assumed that (i) when the positive functional connectivity of target regions with mPFC positively correlated with mindreading scores, mindreading performances would be higher when the activity between the target ROI and mPFC was synchronized, (ii) when the positive functional connectivity negatively correlated with mindreading scores, mindreading performances would be higher when the activity between the target ROI and mPFC was desynchronized, (iii) when the negative functional connectivity negatively correlated with mindreading scores, mindreading performances would be higher when the activity between the target ROI and mPFC was anti-synchronized, and (iv) when the negative functional connectivity positively correlated with mindreading scores, mindreading performances would be higher when the activity between the target ROI and mPFC was more desynchronized (in other words, less antisynchronized). The threshold for significance was set at *P* < 0.05 FDR-corrected.

**Fig. 2:**
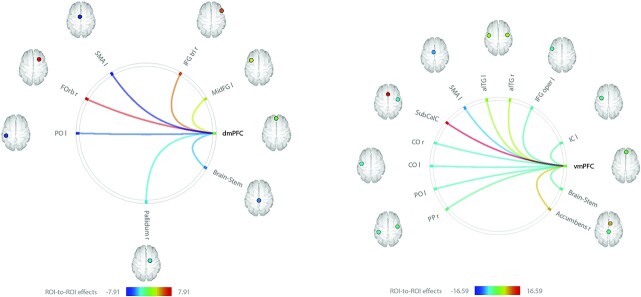
Significant correlations between the BOLD time courses of the ventral and dorsal mPFC and BOLD time courses of brain regions of the Harvard-Oxford atlas. Statistical significance was thresholded at *P* < 0.05 FDR-corrected. Positive correlations are represented by hot colors ranging from yellow to red, and negative correlations by cold colors ranging from green to blue. aITG: Anterior inferior temporal gyrus; CO: Central opercular cortex; dmPFC: Dorsomedial prefrontal cortex; FOrb: Frontal orbital cortex; IC: Insular cortex; IFG oper: Inferior frontal gyrus pars opercularis; IFG tri: Inferior frontal gyrus pars triangularis; MidFG: Middle frontal gyrus; PO: Parietal operculum; PP: Planum polare; SubCalC: Subcallosal cortex; vmPFC: Ventromedial prefrontal cortex; r: Right; l: Left.

## Results

### mPFC functional connectivity related to mindreading scores

The statistical analyses revealed regions whose connectivity with the dmPFC (Fig. 1, left) was positively or negatively correlated with each of the cognitive and the affective mindreading scores (Supplementary Table 1). We observed positive correlations between the cognitive mindreading score and the functional connectivity of the dmPFC (Fig. 1a, left) with (i) the putamen bilaterally (left putamen: *t*(53) = 2.30, *P* = 0.025; right putamen: *t*(53) = 2.61, *P* = 0.012), (ii) left parietal operculum (*t*(53) = 2.61, *P* = 0.012), (iii) right frontal orbital cortex (FOrb; *t*(53) = 2.21, *P* = 0.032), (iv) right IFG pars triangularis (IFG tri; *t*(53) = 2.15, *P* = 0.037), and (v) right pallidum (*t*(53) = 2.01, *P* = 0.050); and a negative correlation between the cognitive mindreading score and the functional connectivity between the dmPFC and the left middle frontal gyrus (MidFG; *t*(53) = −2.09, *P* = 0.041). Regarding affective mindreading (Fig. 1b, left), we observed positive correlations between the score and the functional connectivity of the dmPFC with (i) the right amygdala (*t*(53) = 2.30, *P* = 0.025) and (ii) right FOrb (*t*(53) = 2.01, *P* = 0.050); and negative correlations between the score and the functional connectivity between the dmPFC and (i) the brain-stem (*t*(53) = −2.14, *P* = 0.037), (ii) left IFG pars opercularis (IFG oper; *t*(53) = −2.09, *P* = 0.041), and (iii) left supplementary motor area (SMA; *t*(53) = −2.08, *P* = 0.042).

The statistical analyses also revealed regions whose connectivity with the vmPFC (Fig. 1, right) was positively or negatively correlated with the cognitive and affective mindreading scores (Supplementary Table 1). We observed positive correlations between the cognitive mindreading score and the functional connectivity of the vmPFC (Fig. 1a, right) with (i) the left parietal operculum (*t*(53) = 3.45, *P* = 0.001), (ii) central opercular cortex bilaterally (left central opercular cortex: *t*(53) = 2.25, *P* = 0.029; right central opercular cortex: *t*(53) = 2.39, *P* = 0.021), (iii) right IFG tri (*t*(53) = 2.30, *P* = 0.026), (iv) Heschl’s gyrus bilaterally (left Heschl’s gyrus: *t*(53) = 2.23, *P* = 0.030; right Heschl’s gyrus: *t*(53) = 2.29, *P* = 0.026), (v) thalamus bilaterally (left thalamus: *t*(53) = 2.25, *P* = 0.029; right thalamus: *t*(53) = 2.29, *P* = 0.026), (vi) left insular cortex (IC; *t*(53) = 2.26, *P* = 0.028), (vii) brainstem (*t*(53) = 2.03, *P* = 0.047), and (viii) right planum polare (*t*(53) = 2.01, *P* = 0.050). In addition, we observed negative correlations between this score and the functional connectivity between the vmPFC and the anterior inferior temporal gyrus bilaterally (aITG; left ITG: *t*(53) = −2.06, *P* = 0.045; right aITG: *t*(53) = −2.76, *P* = 0.008) as well as subcallosal cortex (SubCalC; *t*(53) = −2.57, *P* = 0.013). Regarding the affective mindreading score (Fig. 1b, right), we observed a positive correlation between the score and the functional connectivity between the vmPFC and the right posterior superior temporal gyrus (pSTG; *t*(53) = 2.02, *P* = 0.049); and a negative correlation between the score and the functional connectivity between the vmPFC and (i) the left IFG oper (*t*(53) = −2.79, *P* = 0.007), (ii) left SMA (*t*(53) = −2.24, *P* = 0.029), and (iii) right nucleus accumbens (*t*(53) = −2.07, *P* = 0.043). An analysis excluding participants that answered correctly only 3 out of 6 questions on the control questions of the MASC task produced comparable results. None of these correlations survived correction for multiple comparisons.

**Fig. 3:**
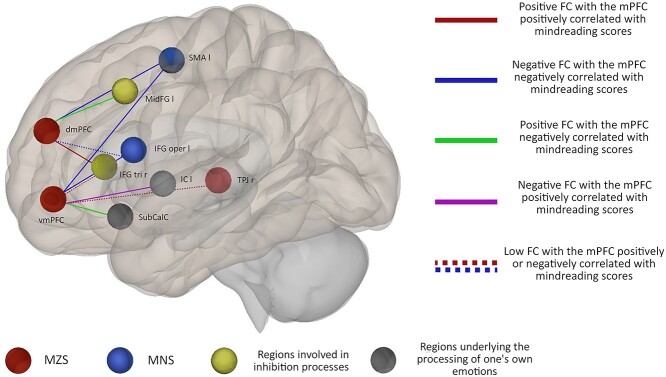
Functional networks related to the dorsal and ventral mPFC and associated with the mindreading processes highlighted in the present study. dmPFC: Dorsomedial prefrontal cortex; IC: Insular cortex; IFG oper: Inferior frontal gyrus pars opercularis; IFG tri: Inferior frontal gyrus pars triangularis; MidFG: Middle frontal gyrus; SubCalC: Subcallosal cortex; vmPFC: Ventromedial prefrontal cortex; r: Right; l: Left; MNS: Mirror neuron system; FC: Functional connectivity.

### Functional connectivity of the mPFC with regions defined by the Harvard-Oxford atlas

We examined the functional connectivity between both the dmPFC and vmPFC and the regions highlighted by the first analysis (Fig. 2, Supplementary Table 2). Regarding the dmPFC (Fig. 2, left), we observed positive correlations between the BOLD time series of this region and those of (i) the right FOrb (*t*(55) = 6.90, *P* < 0.001), (ii) right IFG tri (*t*(55) = 5.37, *P* < 0.001), and (iii) left MidFG (*t*(55) = 2.36, *P* = 0.030) on one hand; and negative correlations between the BOLD time series of the dmPFC and those of (i) the left SMA (*t*(55) = −7.91, *P* < 0.001), (ii) left parietal operculum (*t*(55) = −6.30, *P* < 0.001), (iii) brain-stem (*t*(55) = −4.82, *P* < 0.001), and (iv) right pallidum (*t*(55) = −3.30, *P* = 0.003) in another hand. Regarding the vmPFC (Fig. 2, right), we observed positive correlations between the BOLD time series of this region and those of (i) the SubCalC (*t*(55) = 16.59, *P* < 0.001), (ii) right accumbens (*t*(55) = 7.78, *P* < 0.001), and (iii) aITG bilaterally (left aITG: *t*(55) = 2.85, *P* = 0.009; right aITG: *t*(55) = 3.52, *P* = 0.002) on one hand; and negative correlations between the BOLD time series of this region and those of (i) the left SMA (*t*(55) = −9.17, *P* < 0.001), (ii) central opercular cortex bilaterally (left central opercular cortex: *t*(55) = −5.90, *P* < 0.001; right central opercular cortex: *t*(55) = −5.82, *P* < 0.001), (iii) left parietal operculum (*t*(55) = −5.25, *P* < 0.001), (iv) left IFG oper (*t*(55) = −4.76, *P* < 0.001), (v) left IC (*t*(55) = −4.23, *P* < 0.001), (vi) brain-stem (*t*(55) = −3.54, *P* = 0.001), and (vii) right planum polare (*t*(55) = −3.23, *P* = 0.003) in another hand.

## Discussion

The results highlighted a distinction between the dorsal part of the mPFC connected to the IFG tri and MidFG, regions subtending inhibition processes notably of one’s own perspective (in yellow in Fig. 3); and the ventral part connected to the SubCalC and insula, brain regions involved in internal emotional processes (in gray in Fig. 3). These findings could reflect a role of this region to monitor the salience of one’s own mental contents. Evidence also showed the negative connectivity of the mPFC being part of the MZS (in red in Fig. 3) with regions belonging to the mirror neurons system (MNS) (i.e. the SMA and IFG oper), thought to be involved in the simulation of mental states (in blue in Fig. 3). This suggested a role of the mPFC in the interaction between the MZS and MNS during mindreading.

### Reducing the salience of its own mental content

#### Processing of one’s own perspective

The BOLD time series of the dmPFC and right IFG tri were positively correlated, suggesting these two regions functionally communicate. The positive correlation between this positive connectivity and the performance of attribution of cognitive mental states supports the view that the more functionally connected these regions, the greater the ability to infer the cognitive mental states of others. Consistent with this finding, the pars triangularis part of the right IFG has been shown to be closely involved in the inhibition of one’s own perspective thereby reducing the impact of the individual’s own mental states during the attribution of mental states to others (Van der Meer et al. 2011; Hartwright et al. 2015; Samson et al. 2015). Similarly, we observed a positive functional communication between the dmPFC and MidFG [i.e. dorso lateral prefrontal cortex (dlPFC)], but this one was negatively correlated with the cognitive mindreading performances. In other words, the more the activity of these regions are desynchronized, the better mindreading performances are. Consistent with our results, the dlPFC is considered to be involved in cognitive mindreading (Stuss et al. 2001; Abu-Akel and Shamay-Tsoory 2011). It would be involved in conflict resolution between one’s own and others visual perspectives (Qureshi et al. 2020). Qureshi et al. (2020) suggested that the IFG involved in inhibition of one’s own perspective would act together with the dlPFC allowing the selection between concurrent perspectives. In line with the assumed roles of these regions, our findings suggested that the inhibition of the self-perspective in favor of the perspective of others during mindreading seems to be driven by a desynchronization between the dmPFC and dlPFC activities and the synchronization between the activity of the dmPFC and IFG tri.

#### Processing of one’s own emotions

The BOLD time series of the vmPFC and SubCalC were positively correlated, reflecting the functional communication between the 2 regions. The negative correlation between the functional connectivity of these 2 regions and the cognitive mindreading score could indicate that the less functionally connected these brain areas, the greater the ability to infer the cognitive mental states of others. In other words, it suggested that the activities of these regions need to be desynchronized to enhance mindreading performances. Mindreading researchers are beginning to show interest in the SubCalC (Lockwood et al. 2016; Diaconescu et al. 2017). Its role in mindreading is however currently unclear. Consistent with these results, lesion studies have yielded evidence that this area plays a role in the generation and automatic regulation of negative emotions (George et al. 1995; Drevets et al. 2008; Palomero-Gallagher et al. 2015). Moreover, Hiser and Koenigs (2018) claimed that the interaction between the SubCalC and the vmPFC affects the regulation of emotions. We can assume that the attribution of mental states to others or the observation of social interactions generates feelings in the observer. Moreover, individuals have to extract themselves from their own mental states when attributing mental states to others. We therefore suggest that the desynchronization between the SubCalC and the mPFC makes it possible to maintain a neutral stance, by decoupling one’s own emotional reactions from the attribution of intentions to others, when observing social interactions.

The investigation of relationships between cognitive mindreading and the connectivity of the mPFC highlighted 2 different mechanisms. First, the relationship between the dmPFC and both the IFG tri and dlPFC seemed to play a role to decrease the salience of one’s own perspective during the observation of social interactions. Second, a desynchronization between the vmPFC and the SubCalC activities seems to be necessary to individuals to set aside the emotional states caused by watching social interactions. Altogether, these findings point the involvement of the ventral and dorsal mPFC and their connectivities to keep a neutral stance by the decreased salience of the individual’s own mental state during the attribution of mental states to others.

### Two antisynchronized systems

The correlation analysis between the functional connectivity of the ventral and dorsal mPFC and affective mindreading performances highlighted 2 different networks, the MZS of which the mPFC and pSTG (i.e. the TPJ) are being part; and the MNS including the SMA and IFG oper.

#### Mentalizing system

The positive correlation between the affective mindreading performances and the connectivity between the vmPFC and right pSTG, which is part of the TPJ (Schurz et al. 2017), confirmed the role of this parietal region in mentalizing. Owing to its specific involvement in social versus nonsocial inferences, the TPJ was assumed to be crucial for general reasoning about mental states (Saxe and Kanwisher 2003; Saxe and Wexler 2005). In line with its involvement in attentional processes, it was suggested that the TPJ also plays a role in switching between the self and others’ perspective (Corbetta et al. 2008). Our results are consistent with the assumed role of these regions and with Samson’s model, in which the mPFC and TPJ together underlie mental state attribution based on reasoning, using semantic knowledge and more particularly information about social concepts and rules, allowing attention to be oriented toward relevant environmental stimuli (Samson 2009).

#### Simulation system

Our results also evidenced a role of a network characterized by a negative correlation with the BOLD time series of the ventral and dorsal mPFC. It has been suggested that negative correlations between the BOLD time series of 2 regions represent either a segregation of neural processes subserving opposite goals in different brain regions (Fox et al. 2005) or integration mechanisms for sharing and processing information (Hampson et al. 2010). In addition, negative connectivity could reflect antisynchrony between 2 regions. Both the ventral and dorsal mPFC on one hand and the SMA, and IFG oper on another hand, could thus belong to 2 distinct antisynchronized networks whose BOLD time series were negatively correlated. There was a negative correlation between this negative connectivity and the mindreading performances, such that the more negatively connected these regions, the better the mindreading performances. The relationship between the connectivity of the IFG oper and SMA and the mindreading abilities is consistent with previous findings, as they all belong to the MNS, a set of brain regions containing neurons that are activated by the performance of an action, but also by the observation of others performing this same action (Rizzolatti et al. 2001; Caspers et al. 2010). This network was initially assumed to subtend the comprehension of intentions via simulating observed motor actions (Gallese and Goldman 1998) and subsequently expanded to the simulation of the emotions of others, notably through the recognition of facial expressions (de Vignemont and Singer 2006; Iacoboni and Dapretto 2006; Keysers and Gazzola 2007).

#### Interactions in antisynchrony

Our findings highlighted the relationship between 2 distinct networks associated with affective mental state attribution: the MZS containing the mPFC, and the MNS whose interaction in antisynchrony with the mPFC seemed to be related with mindreading, as suggested by the negative connectivity between the dorsal and ventral mPFC and regions belonging to the MNS. Numerous hybrid models of mindreading have postulated the existence of opposite systems based on the MNS, subtending simulation processes, and the MZS, for reasoning on mental states (Keysers and Gazzola 2007; Samson 2009; Shamay-Tsoory et al. 2009; Van Overwalle and Baetens 2009). We can therefore assume that the MZS and MNS, respectively, subtend processes involved in reasoning and simulation and have to be coordinated in antisynchrony to lead to efficient mindreading performances. Consistent with the idea of antisynchrony, Samson (2009) claimed that there is a balance between the involvement of these 2 systems.

Although they have yet to be confirmed, several assumptions have been made about the regions responsible for the interaction between the MNS and the MZS. For example, authors have discussed the role played by the connectivity between the mPFC and the IFG (Uddin et al. 2007; Spunt and Lieberman 2012; Sperduti et al. 2014), precuneus and inferior parietal lobule (Uddin et al. 2007), or insula (Keysers and Gazzola 2007). Without excluding a potential role of other regions in this interaction, our results support the role of the dorsal and ventral mPFC in establishing a link between these systems via the IFG oper and SMA.

### Functional distinction between dmPFC and vmPFC

In relation with mindreading, the dorsal and ventral mPFC showed common functional connectivities including the antisynchrony with the MNS. This result corresponded to previous findings showing interactions between the MNS and mPFC without specific distinctions between the dorsal and ventral parts (Spunt and Lieberman 2012; Sperduti et al. 2014). Nonetheless, as discussed above, the dorsal and ventral mPFC also showed dissimilar connectivities. The negative connectivity of the vmPFC with insula and the surrounding operculum was positively correlated with cognitive mindreading performances indicating that the more these regions were desynchronized the better the cognitive mindreading performances were; a kind of correlation whose interpretation is difficult. Yet, it has been argued that these insular regions would be specifically involved in affective mindreading (Corradi-Dell’Acqua et al. 2020) by subtending affective resonance mechanisms through which physiological and behavioral reactions could be simulated on oneself (Shamay-Tsoory 2011; Stietz et al. 2019). In turn, the functional connectivity between the dmPFC and FOrb was positively correlated with cognitive and affective mindreading abilities. Namely, the more synchronized these regions, the better the mindreading performances. Amodio and Frith (2006) suggested that the FOrb could guide behavior in terms of possible outcome values, as opposed to the dmPFC which would guide behavior in terms of possible futures actions values. Consistent with Amodio and Frith (2006) proposal, our results highlighted a dialogue between the dmPFC and FOrb guiding future decision-making and behavior.

In sum, as hypothesized, a functional ventro-dorsal distinction was found within the mPFC. The vmPFC showed connectivities with insular and subcallosal regions closely linked with self-emotional processes. Consistent with Li et al. (2014) proposal, these connectivities were associated with emotional engagement. In contrast, connectivities between the dmPFC and the IFG tri and dlPFC which underlie the inhibition of one’s own perspective as well as the FOrb involved in decision-making are consistent with previous evidence about the role of the dmPFC in higher level executive processes (Amodio and Frith 2006; D’Argembeau et al. 2007; Isoda and Noritake 2013). Our study helped to disentangle the functional connectivities underlying the differential functions of the dorsal and ventral mPFC. In line with this distinction, we propose a role of the connectivity of the ventral and dorsal mPFC to respectively decrease the salience of one’s own affective and cognitive mental contents during the attribution of cognitive mental states.

### Features of the task used and limitations

Some of the features of the MASC task used in this study seem to explain the particular results highlighted here. The video format of the MASC task makes it possible to observe the dynamics of facial and gestural expressions and goal-directed actions, and thus to attribute mental states through simulation mechanisms. This may explain why the MNS was highlighted here, but rarely in other studies, which mostly used tasks featuring stimuli without a dynamic dimension. Besides the ability to inhibit one’s own perspective, our study showed the importance of being able to extract oneself from one’s own emotional states, in order to attribute mental states to others. This may be related to the task feature explained above and the presence of a social context, which encourages participants to involve themselves in the movie. Ibanez and Manes (2012) had already highlighted the importance of internal representations in contextualized social interactions, suggesting that a network is responsible for integrating internal and external states triggered by contextual cues in social situations. Although participants in the present study were only observing social interactions, our findings highlight the need to further investigate mindreading processes in tasks reflecting everyday social interactions in which participants are directly involved.

Our use of resting-state fMRI means that these findings should be interpreted with caution. Although recent studies suggest that networks spatial organization may dynamically fluctuate over time (Allen et al. 2014; Ciric et al. 2017), in order to simplify the interpretation, our analyses were based on the assumption of stable functional connectivity over time. Moreover, the fact that the MRI session and the MASC task took place separately resulted in indirect measures, leading to a lack of insight into the cognitive processes engaged during the MRI session. However, findings based on questionnaires and neuroimaging data suggest that mindreading is a cognitive process that take place during rest (Schilbach et al. 2008; Diaz et al. 2013). Another limitation arising from the use of this technique is the lack of statistical power, which prevented us from correcting for multiple comparisons in order to address the false positives issue. The latter can result from the noise that systematically accompanies resting-state fMRI data (Krüger and Glover 2001; Liu et al. 2006).

## Conclusion

The results highlighted the connectivity of the mPFC with regions involved in the regulation of the salience of one’s own mental contents. Consistent with the literature, a functional distinction was demonstrated with the dorsal part of the mPFC connected to the IFG tri and dlPFC, regions subtending inhibition of one’s own perspective, and the ventral part connected to the SubCalC and insula, brain regions involved in emotional processes. Overall, this evidence emphasized a role of the mPFC to keep a neutral stance by decreasing the salience of one’s own mental content. The findings also highlighted 2 anticorrelated networks, the MZS including the mPFC, and the MNS, involved in the simulation of mental states. This suggested a role of the mPFC in the antisynchronous interaction of the MZS and the MNS, subtending a balance between reasoning and simulation processes. The task used in this study using social situations that were closer to real life than other traditional tasks could account for the evidence of complex interactions in relation to the mPFC. It highlights the need to investigate mindreading processes in tasks that directly involve participants.

## Supplementary Material

table_supp_tgac032
